# Wellness Coaching for People With Prediabetes: A Randomized Encouragement Trial to Evaluate Outreach Methods at Kaiser Permanente, Northern California, 2013

**DOI:** 10.5888/pcd12.150251

**Published:** 2015-11-25

**Authors:** Hong Xiao, Sara R. Adams, Nancy Goler, Rashel S. Sanna, Mindy Boccio, David J. Bellamy, Susan D. Brown, Romain S. Neugebauer, Assiamira Ferrara, Julie A. Schmittdiel

**Affiliations:** Author Affiliations: Hong Xiao, Sara R. Adams, Susan D. Brown, Romain S. Neugebauer, Assiamira Ferrara, Division of Research, Kaiser Permanente Northern California, Oakland, California; Nancy Goler, David J. Bellamy, Wellness Coaching Center, Kaiser Permanente Northern California, Vallejo, California; Rashel S. Sanna, Mindy Boccio, Regional Health Education, Kaiser Permanente Northern California, Oakland, California.

## Abstract

**Introduction:**

Health coaching can improve lifestyle behaviors known to prevent or manage chronic conditions. Little is known about effective ways to encourage health and wellness coaching among people who might benefit. The purpose of this randomized encouragement trial was to assess the relative success of 3 outreach methods (secured email message, telephone message, and mailed letter) on the use of wellness coaching by people with prediabetes.

**Methods:**

A total of 14,584 Kaiser Permanente Northern California (KPNC) patients with diagnosed prediabetes (fasting plasma glucose, 110–125mg/dL) were randomly assigned to be contacted via 1 of 4 intervention arms from January through May 2013. The uptake rate (making an appointment at the Wellness Coaching Center [WCC]) was assessed, and the association between uptake rate and patient characteristics was examined via multivariable logistic regression.

**Results:**

The overall uptake rate across intervention arms was 1.9%. Secured email message had the highest uptake rate (3.0%), followed by letters and telephone messages (*P* < .05 for all pairwise comparisons). No participants in the usual-care arm (ie, no outreach) made an appointment with the WCC. For each year of increased age, the estimated odds of the uptake increased by 1.02 (odds ratio [OR] = 1.02; 95% CI, 1.01–1.04). Women were nearly twice as likely to make an appointment at the WCC as men (OR = 1.87; 95% CI, 1.40–2.51).

**Conclusion:**

Our results suggest that the WCC can recruit and encourage KPNC members with prediabetes to participate in the WCC. Future research should focus on increasing participation rates in health coaching among patients who may benefit.

## Introduction

In 2012, 86 million Americans aged 20 years or older had prediabetes (1). Studies suggest that people with prediabetes have a high risk for developing type 2 diabetes in 5 years if they do not receive appropriate prevention interventions (2,3). The Diabetes Prevention Program study reported that a 58% reduction in incidence of type 2 diabetes was observed among adults with prediabetes during the 3-year follow-up as a result of lifestyle interventions to improve diet, increase physical activity, and encourage weight loss (4). Effective lifestyle interventions help prevent type 2 diabetes from developing among people with prediabetes (4,5).

Health coaching, often delivered by nonphysician health care providers, enhances patients’ commitment to lifestyle change via evidence-based behavioral change techniques, such as motivational interviewing (6–8). Studies suggest that health coaching improves compliance and outcomes for patients with chronic diseases (7,9) and reduces medical costs and hospitalizations (10). These types of health and wellness coaching services may be even more effective when fully integrated into a person’s overall health care delivery system (11).

Despite the potential for wellness coaching to improve lifestyle behaviors for patients with prediabetes and to delay or prevent the onset of diabetes, little is known about optimal approaches to encourage the uptake of wellness coaching in populations of people with prediabetes. This evaluation was conducted as part of the Natural Experiments for Translation in Diabetes Study, which tests the effectiveness of population-targeted diabetes prevention and control policies. The goal of this randomized encouragement trial was to examine the effectiveness of different methods of encouraging patients with prediabetes to use the Wellness Coaching Center (WCC) at Kaiser Permanente Northern California (KPNC). We hypothesized that 3 outreach interventions (secured email message, telephone message, and letter) would increase the uptake of wellness coaching among KPNC patients with prediabetes.

## Methods

### Setting

KPNC is a large, integrated health care delivery system currently serving approximately 3.5 million members in Northern California. The KPNC membership is diverse, community-based, and broadly representative of the local and statewide population. Since 2010, KPNC provided a health coaching program via the WCC that helps patients’ make lifestyle changes to reduce diabetes risk: eating more healthfully, increasing physical activity, achieving and maintaining healthy weight, quitting smoking, and reducing stress. Wellness coaching is a free service for KPNC members. Members are connected with the WCC program through referrals by KPNC health care providers and staff, partnership with employers, self-referral, and outreach through the medical facility (12,13).

### Participants

Patients were eligible for the study if they were an active KPNC member aged 18 to 80 years, lived in California, spoke English, and had a fasting plasma glucose from 110 to 125 mg/dl (prediabetes or impaired fasting glucose as defined by the World Health Organization) within 6 months before the study start date. In addition, eligible patients were excluded if they participated in the KPNC WCC program previously. Patients were excluded if they had any 1 of the following conditions: an acute myocardial infarction in the previous year, corticosteroid use in the previous year, pregnancy, or a diagnosis of diabetes, dementia, or cancer. These patients were excluded because they may have had high fasting plasma glucose values for reasons other than prediabetes or may have been too ill to participate in the WCC program.

### Study design

This study was a randomized encouragement trial with outcome data collected prospectively. Randomization was first stratified on the basis of whether patients were active on www.kp.org, the KPNC electronic patient portal, because only members who are active on www.kp.org can receive secure email messages. Active patients were defined as those who had registered on www.kp.org, had agreed to receive emails, and had logged on within the past 18 months. Participants were divided into 2 cohorts: secure-message-eligible members and secure-message-ineligible members.

Secure-message-eligible members were randomized into 1 of 4 arms: Arm A received a secured email message, Arm B received an interactive voice response (IVR) telephone message, Arm C received a mailed letter, and Arm D received no active study outreach (ie, usual care). Secure-message–ineligible members were randomized into 1 of 3 arms: Arm B, Arm C, and Arm D (Figure). Patients in the usual-care arm received no specific encouragement from this randomized encouragement trial to participate in the WCC program, but they could have been referred to the WCC program via the usual methods (ie, through their primary care physician or clinical staff or through a facility-based or employer-based outreach program). Randomization status was determined by assigning each person a random number using SAS random number generator (SAS Corp, version 9.1.3). The data set was then sorted by the random number. For secure-message-eligible members, the first 25% were assigned to Arm A, the secured email message arm; the second 25% to Arm C, the letter arm; the third 25% to the Arm B, the IVR telephone message arm; and the fourth 25% to Arm D, usual care. For secure-message-ineligible members, the first 33% were assigned to Arm C, the letter arm; the second 33% to Arm B, the IVR telephone message arm; and the final 33% were assigned to Arm D, usual care.

**Figure:**
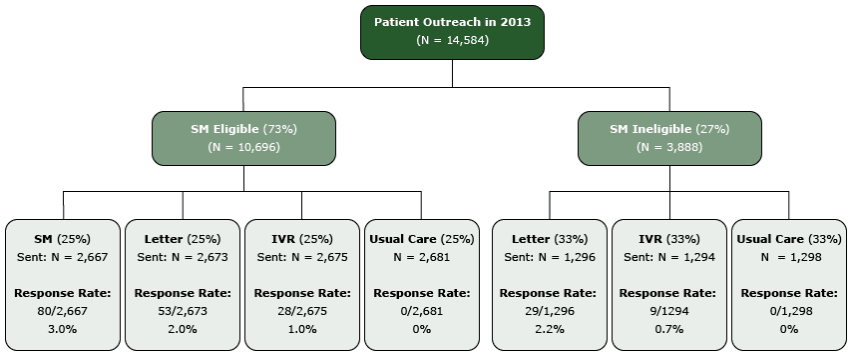
Uptake to the Kaiser Permanente Northern California’s Wellness Coaching Center (WCC) by intervention arm. All pairwise comparisons were significant at *P *.02 (2 by 2 Pearson χ^2^ test). Abbreviations: SM, secure messaging; IVR, interactive voice response.

Outreach materials were sent out from January through May, 2013. All messages introduced the WCC with contact information and emphasized that people were often more successful and maintained healthy changes when they received support along the way, which is particularly important to those who have a higher than normal (fasting plasma glucose 110–125 mg/dl) blood glucose level. All outreach messages were the same across study intervention arms (with the exception of usual-care patients, who received no message); the only difference was the method by which the messages were sent.

After randomization and before outreach, primary care physicians were contacted and given the opportunity to opt out of having their patients participate in the study; physicians excluded only 28 (0.3%) patients from the study.

### Data collection

All data for this study were derived from KPNC electronic health records and administrative databases: patient age, birth year, sex, race/ethnicity, laboratory results, diagnostic data, and appointment information.

### Outcome measures

Our study’s main outcome measure was the uptake rate for WCC participation, which was measured by whether patients made an appointment with the WCC within 6 weeks after they were contacted via any one of study intervention methods (secure email message, IVR telephone message, letter). We also assessed whether those in the usual care arm (who received no study contact) made a WCC appointment.

### Statistical analysis

Initial descriptive statistical analyses were conducted for the outcome (the uptake of WCC) and independent variables, which were age, sex, race/ethnicity, fasting plasma glucose level, body mass index (BMI) (kg/m2), and primary care visit counts at baseline. We used χ^2^tests to compare categorical variables. Multivariable logistic regression was used to analyze the independent predictors of the outcome and to calculate odds ratios and 95% confidence intervals for assessing the association between the outcome (the uptake) and independent variables. We designed the study to have 80% power to evaluate the effectiveness of each arm, stratified by secure-message eligibility and with no multiple hypotheses testing, to detect a significant difference at the *P* < .05 level. All analyses were conducted using SAS, version 9.1.3. The KPNC Institutional Review Board approved this study. 

## Results

Overall, 14,584 KPNC members with prediabetes (8,712 [60%] men and 5,872 [40%] women) met study eligibility criteria and were included in the analysis. The mean age was 59 years, and 62% of participants were aged 50 to 69 years. Forty-three percent were obese, and 35% were overweight. Fifty-seven percent were white (Table 1). There were no differences in baseline characteristics across the randomized study arms (Table 2, Table 3).

**Table 1:** Demographic and Clinical Characteristics, Study of Patients (N = 14,584) With Prediabetes, Kaiser Permanente Northern California 2013

Characteristic	N (%)
Age, Y, mean (SD)	59.0 (11.2)
**Age categories, y**	
18–39	798 (5.5)
40–49	2,040 (14.0)
50–59	4,308 (29.5)
60–69	4,735 (32.5)
70–80	2,703 (18.5)
**Sex**	
Female	5,872 (40.3)
Male	8,712 (59.7)
**Race/ethnicity**	
American Indian/Alaska Native	67 (0.5)
Asian	2,787 (19.1)
Black or African American	803 (5.5)
Hispanic	1,702 (11.7)
Native Hawaiian or other Pacific Islander	105 (0.7)
White	8,313 (57.0)
Unknown	807 (5.5)
Body mass index (BMI) (kg/m2), mean, SD	30.5 (6.3)
**BMI categories**	
<25 (Normal)	2,242 (15.4)
25–29 (Overweight)	5,076 (34.8)
≥30 (Obese)	6,258 (42.9)
Unknown	1,008 (6.9)
**Fasting plasma glucose categories, mg/dL**	
110–114	7,790 (53.4)
115–119	4,210 (28.9)
120–125	2,584 (17.7)
Patients with diagnosis of prediabetes at enrollment	9,007 (61.8)
**Number of primary care visits in previous year**	
0	774 (5.3)
1	3,769 (25.8)
2	3,493 (24.0)
3	2,472 (16.9)
≥4	4,076 (28.0)

Abbreviations: BMI, body mass index; SD, standard deviation.

**Table 2:** Demographic and Clinical Characteristics, Study of Patients (N = 14,584) With Prediabetes, Secure-Messaging-Eligible Cohort Stratified by Arm^a^, Kaiser Permanente Northern California 2013

Characteristic	Study Arm				*P* Value^b^
	A. Secure Email Message, N (%)	B. Interactive Voice Response, N (%)	C. Mailed Letter, N (%)	D. Usual Care (No Contact), N (%)	
**N**	2,667	2,675	2,673	2,681	NA
**Age, mean (SD)**	58.3 (11.2)	58.2 (11.1)	58.4 (11.1)	58.2 (10.9)	.92
**Age categories**					
18–39	177 (7)	167 (6)	157 (6)	153 (6)	.24
40–49	384 (14)	391 (15)	391 (15)	392 (15)	
50–59	769 (29)	785 (29)	828 (31)	842 (31)	
60–69	914 (34)	929 (35)	852 (32)	907 (34)	
70–80	423 (16)	403 (15)	445 (17)	387 (14)	
**Sex**					
Female	1,077 (40)	1,084 (41)	1,029 (38)	1,057 (39)	.39
Male	1,590 (60)	1,591 (59)	1,644 (62)	1,624 (61)	
**Race/ethnicity**					
American Indian/Alaska Native	5 (0.2)	16 (0.6)	13 (0.5)	13 (0.5)	.75
Asian	517 (19)	473 (18)	490 (18)	501 (19)	
African American	115 (4)	106 (4)	98 (4)	116 (4)	
Hispanic	242 (9)	262 (10)	254 (10)	259 (10)	
Native Hawaiian or other Pacific Islander	21 (1)	15 (1)	17 (1)	14 (1)	
White	1,642 (62)	1,662 (62)	1,668 (62)	1,644 (61)	
Unknown	125 (5)	141 (5)	133 (5)	134 (5)	
**BMI (kg/m2) mean, SD**	30.6 (6.4)	30.6 (6.2)	30.6 (6.1)	30.6 (6.5)	.97
**BMI categories (kg/m2)**					
<25 (Normal)	409 (15)	407 (15)	377 (14)	423 (16)	.63
25–29 (Overweight)	930 (35)	934 (35)	895 (33)	909 (34)	
≥30 (Obese)	1,132 (42)	1,151 (43)	1,198 (45)	1,149 (43)	
Unknown	196 (7)	183 (7)	203 (8)	200 (7)	
**Fasting plasma glucose categories, mg/dL**					
110–114	1,465 (55)	1,445 (54)	1,382 (52)	1,446 (54)	.26
115–119	733 (27)	761 (28)	817 (31)	758 (28)	
120–125	469 (18)	469 (18)	474 (18)	477 (18)	
**Prediabetes diagnosis at enrollment**	1,657 (62)	1,665 (62)	1,662 (62)	1,668 (62)	>.99
**Number of primary care visits in previous year**					
0	149 (6)	145 (5)	161 (6)	153 (6)	.89
1	663 (25)	704 (26)	676 (25)	683 (25)	
2	662 (25)	612 (23)	651 (24)	641 (24)	
3	464 (17)	441 (16)	452 (17)	455 (17)	
≥4	729 (27)	773 (29)	733 (27)	749 (28)	

Abbreviation: SD, standard deviation.

a Type of communication used to encourage participants to participate in Kaiser Permanente’s Wellness Coaching Center.

b *P* values calculated with χ^2^ and *t* tests.

**Table 3:** Demographic and Clinical Characteristics, Study of Patients (N = 14,584) With Prediabetes, Secure-Messaging-Ineligible Cohort Stratified by Arm^a^, Kaiser Permanente Northern California, 2013

Characteristic	Study Arm			*P* Value^b^
	B. Interactive Voice Response, N (%)	C. Letter, N (%)	D. Usual Care (No Contact), N (%)	
**N**	1,294	1,296	1,298	NA
**Age, mean (SD)**	60.9 (11.4)	61.2 (11.3)	60.9 (11.7)	.78
**Age categories, y**				
18–39	43 (3)	44 (3)	57 (4)	.27
40–49	173 (13)	161 (12)	148 (11)	
50–59	362 (28)	345 (27)	377 (29)	
60–69	373 (29)	405 (31)	355 (27)	
70–80	343 (27)	341 (26)	361 (28)	
**Sex**				
Female	545 (42)	535 (41)	545 (42)	.90
Male	749 (58)	761 (59)	753 (58)	
**Race/ethnicity**				
American Indian/Alaska Native	9 (0.7)	6 (0.5)	5 (0.4)	.45
Asian	289 (22)	247 (19)	270 (21)	
Black or African American	111 (9)	118 (9)	139 (11)	
Hispanic	213 (16)	235 (18)	237 (18)	
Native Hawaiian or Other Pacific Islander	11 (1)	13 (1)	14 (1)	
White	567 (44)	588 (45)	542 (42)	
Unknown	94 (7)	89 (7)	91 (7)	
**BMI (kg/m2), mean, SD**	30.2 (6.0)	30.4 (6.2)	30.2 (6.2)	.78
**BMI categories**				
<25 (Normal)	217 (17)	198 (15)	211 (16)	.87
25–29 (Overweight)	459 (35)	486 (38)	463 (36)	
≥30 (Obese)	543 (42)	533 (41)	552 (43)	
Unknown	75 (6)	79 (6)	72 (6)	
**Fasting plasma glucose categories, mg/dL**				
110–114	698 (54)	689 (53)	665 (51)	.46
115–119	377 (29)	383 (30)	381 (29)	
120–125	219 (17)	224 (17)	252 (19)	
**Prediabetes diagnosis at enrollment**	784 (61)	785 (61)	786 (61)	> .99
**Number of primary care visits in previous year**				
0	61 (5)	51 (4)	54 (4)	.59
1	352 (27)	353 (27)	338 (26)	
2	324 (25)	296 (23)	307 (24)	
3	210 (16)	236 (18)	214 (16)	
≥4	347 (27)	360 (28)	385 (30)	

Abbreviation: SD-standard deviation.

a Type of communication used to encourage participants to participate in Kaiser Permanente’s Wellness Coaching Center

b *P* values calculated with χ^2^ and *t* tests.

The overall uptake rate across intervention arms was 1.9% (199/10,605). There was no uptake among usual-care patients (ie, no patients in this arm made an appointment with the WCC within 6 weeks of outreach). The secured email message had the highest uptake rate, 3.0% (80/2667). For letter recipients, uptake was 2.0% among secure-message-eligible members, and 2.2% among secure-message-ineligible members; for IVR telephone message recipients, uptake was 1.0% among secure-message-eligible members, and 0.7% among secure-message-ineligible members (Figure).

Each intervention arm had a higher uptake rate than the usual-care arm. Among secure-message-eligible members, the secured email message had higher uptake rate than the letter, which had a higher uptake rate than the IVR telephone message (*P* <.05 for all pairwise comparisons). Among secure-message–ineligible members, the letter also had a higher uptake rate than the IVR telephone message.

Patient age was associated with uptake of the WCC program. For each additional year of age, the estimated odds of making an appointment increased significantly (OR = 1.02; 95% CI, 1.01–1.04). Women were nearly twice likely to make an appointment than men (OR = 1.87; 95% CI, 1.40–2.51). There were no significant differences in uptake between non-Hispanic white patients and African American, Asian, Hispanic, or Native American patients (Table 4).

**Table 4:** Association Between Uptake at Wellness Coaching Center and Independent Variables,^a^ Study of Patients (N = 14,584) With Prediabetes, Kaiser Permanente Northern California, 2013

Variable	Odds Ratio (95% Confidence Interval)
**Body mass index^b^**	0.98 (0.96–1.01)
**Number of primary care visits**	1.02 (0.98–1.06)
**Fasting plasma glucose level^c^**	1.02 (0.99–1.06)
**Age**	1.02 (1.01–1.04)
**Sex**	
**Female (male as referent)**	1.87 (1.40–2.51)
**Race (white as referent)**	
Asian	0.70 (0.45–1.09)
African American	1.86 (0.96–2.64)
Hispanic	0.70 (0.47–1.18)
American Indian/ Alaska Native	1.00 (0.14–7.31)
Unknown	0.56 (0.23–1.37))

a Logistic regression model.

b Calculated as kg of body weight/height in m^2^.

c Measured as mg/dL

## Discussion

The goal of this randomized encouragement trial and analysis was to examine the effectiveness of different methods for encouraging people with prediabetes to use the WCC program at KPNC. Patient uptake (making an appointment with the WCC) was positively correlated with age and sex but was not associated with BMI, number of primary care visits, or fasting plasma glucose level.

Our study found that a secured email message was the best method among patients with email access to increase wellness coaching uptake. Previous studies found that surveys may increase response rates by using email (14,15); our findings suggest that email also may be an effective tool for increasing rates of prevention-program use. Furthermore, each intervention arm had higher update rates than the usual-care arm, which means the intervention arms (secured email message, IVR telephone message, and letter) did bring patients to the WCC. The overall uptake rate across intervention arms was 1.9%. Although this rate may seem low, it is similar to other estimates of response to low-intensity, wide-reach interventions conducted in the general population. For example, in 2012, the Direct Marketing Association reported that direct mail response rates were 3.4%, paid research was 0.22%, and email's average participation rate was only 0.12% (16). In addition, another study examining population participation rates in clinical trials suggests these rates are also low: Kehl et al reported that rates of clinical trial participation by adult cancer populations were below 5%, which is similar to what we found in this study. (17). The uptake rate for WCC participation by the usual-care arm of this study (no outreach) was zero, which shows that even though the uptake rate was relatively low, it still was effective compared with no outreach to patients with prediabetes. The uptake rate across the 3 intervention arms resulted in close to 200 patients with prediabetes accessing the WCC within 6 weeks. Future efforts to reach a large percentage of patients with prediabetes or other high risk patients could result in thousands of initiated coaching sessions.

We also found that women were more likely than men to make an appointment with the WCC. Previous research reported that participants in the KPNC WCC were predominantly women (12,13); another study of health coaching to improve hypertension also had more women participants (64%) than men (18). Our study results also showed that the younger the participants, the less likely they were to make an appointment at WCC. Petter and colleagues reported that young adults aged 18 to 38 were less willing (had lower intention) to participate in a lifestyle intervention than adults aged 39 to 65 (19).These findings suggest that tailoring both the methods and content of outreach to young patients, particularly young men, is an important component to improving their rates of participation in healthy lifestyle programs such as wellness coaching.

Our study has some limitations. First, the outreach methods we tested were low-intensity by design; we do not know how a more intensive outreach with multiple contacts would improve uptake. Heberlein and Baugartner reported that multiple follow-ups via different contact methods could yield higher participation rates than 1-time reminders (20,21). Such multiple contact methods increase the perceived personal relevance and persuasiveness of an intervention (22). In addition, it was beyond the scope of this study to do a formal cost-effectiveness analysis comparing each of the methods. However, although email was the most effective outreach method, it is also likely to be less expensive than regular mail, which requires printing and postage. Finally, this study focused on patients who sought treatment at KPNC, and results might not be generalizable to other groups.

Health coaching is one population-based approach to encouraging healthy lifestyle behaviors. Evidence-based outreach methods are needed to encourage uptake by patients at risk for chronic disease. Our results suggest that an active outreach strategy — secured email message for patients with electronic access and letters for patients without such access — can effectively improve the uptake of wellness coaching by patients with prediabetes. Health care systems should consider outreach strategies to target people who would benefit from coaching to improve healthy lifestyles and reduce disease risk.
